# Prognostic Significance of Regulatory CD25+ T Cells in Bladder Cancer: An Immunohistochemical Analysis

**DOI:** 10.5146/tjpath.2025.13848

**Published:** 2025-09-30

**Authors:** Sarra Ben Rejeb, Nadia Kouki, Hassen Khouni, Rami Boulma, Khadija Bellil

**Affiliations:** Department of Pathology, Security Forces Hospital, Tunis, Tunisia; Department of Urology, Security Forces Hospital, Tunis, Tunisia

**Keywords:** CD25, T cells, Bladder, Cancer

## Abstract

***Objective: *** Tumor-infiltrating lymphocytes (TILs) have been shown to predict outcomes in several cancers. This study aimed to evaluate the density and location of regulatory T cells (Tregs) using immunohistochemistry (IHC) in urothelial carcinomas (UC) of the bladder and to assess their prognostic value.

***Material and Methods:*** We have retrospectively collected all cases of UC of the bladder infiltrating at least the lamina propria, diagnosed in our pathology department between 2011-2021. Specimens were stained for CD3 and CD25. TILs were assessed separately in the tumor core and the stroma. The median TIL count was used as a cut-off to classify cases into low- or high-density groups.

***Results: *** A total of 30 cases were included in the study. The median age of the patients was 65 years, with a male-to-female ratio of 9:1. The distribution of TILs was heterogeneous across locations among patients. CD3+ (p=0.035) and CD25+ (p=0.051) TILs were predominantly observed in the stroma. The density of CD25+ and CD3+ TILs were not associated with clinicopathological features. Multivariate analysis revealed that advanced histological stage and a high density of regulatory CD25+ T lymphocytes were predictive factors of poorer event-free survival (respectively p=0.041 and p=0.052).

***Conclusion:*** Regulatory T cells appear to predict worse survival outcomes. Further studies are needed to confirm their prognostic value.

## INTRODUCTION

Bladder cancer (BC) ranks as the 9th most common malignancy worldwide and is the ninth leading cause of cancer-related mortality in men (1–3). The predominant histological subtype, urothelial carcinoma (UC), is characterized by high recurrence and progression rates (4). At diagnosis, nearly 70% of patients present with non-muscle-invasive bladder cancer (NMIBC) (2,5). Management of NMIBC primarily involves transurethral resection of the bladder tumor (TURBT) followed by intravesical Bacille Calmette-Guérin (BCG) therapy. The five-year progression rate to muscle-invasive bladder cancer (MIBC) ranges from 10% to 30% (2,5), which requires more aggressive treatment approach such as radical cystectomy combined with neoadjuvant chemotherapy (CT). Despite advancements in therapy and close monitoring, BC remains unpredictable in its clinical course, with heterogeneous treatment responses. Moreover, current risk stratification data based on tumor stage and grade often proves to be insufficient to guide optimal therapeutic management, underscoring the need for novel biomarkers.

In this context, it has been established that the immune system plays a key role in cancer, both promoting and suppressing tumor development (6,7). This phenomenon, referred to as cancer immunoediting (7), occurs within the tumor microenvironment (TME). Tumor-infiltrating lymphocytes (TILs) are a critical component influencing tumor progression. Their composition, distribution, and density, collectively referred to as the “immune contexture”, vary across tumors and significantly modulate the host’s anti-tumor response (8). Regulatory T cells (Tregs), a subset of CD3+CD4+ cells, contribute to an immunosuppressive microenvironment by inhibiting effector B and T cells (8). The prognostic significance of Tregs has been investigated in various solid tumors, where they are generally associated with poor outcomes (9,10). However, studies exploring the prognostic role of Tregs in UC remain limited and yield conflicting results (4).

In this study, we aimed to evaluate the density and distribution of Tregs in UC of the bladder using immunohistochemistry (IHC) and to assess their prognostic significance.

## MATERIALS and METHODS

### Patients and Tissue Samples

This was a retrospective, bicentric cross-sectional study approved by the Biomedical Research Ethics Committee of our institution (Approval number 08/23). We collected all tumor samples of UC of the bladder infiltrating at least the lamina propria from patients diagnosed at our pathology department between 2011-2021. Formalin-fixed paraffin-embedded (FFPE) tissues from initial TURBT or cystectomy specimens, whether from treated or de novo cases, were included when available.

Patients with UC confined to the lamina propria, other histological subtypes of bladder cancer, or isolated UC of the upper urinary tract were excluded. Additionally, cases with incomplete clinical records, small or non-representative biopsy samples (mainly involving only the superficial component of the tumor), and specimens difficult to interpret due to extensive necrosis or electrocautery artifacts were excluded.

Clinical data, including age, gender, multifocality, tumor size, treatment, progression, recurrence, metastasis, and survival, were extracted from the patients’ medical records. Pathological data, such as histological subtype, grade, lymphovascular invasion, the presence of in situ carcinoma (ISC), and TNM stage, were retrieved from pathology reports.

### Immunohistochemical Study

#### Protocol

All hematoxylin-eosin-stained sections were initially reviewed to select the FFPE blocks containing samples with adequate inflammatory infiltrate and sufficient tumor material. IHC staining was performed using an automated immunostainer (Leica Bond Max) and monoclonal antibodies against CD25 (clone 4C8, Leica) and CD3 (clone LN10, Leica), following the manufacturer’s protocol.

For each case, 5-micrometer-thick sections were prepared from the selected FFPE blocks. Tissue sections were deparaffinized in xylene and rehydrated through a graded alcohol series, followed by heat-based antigen retrieval in EDTA buffer (pH 8.8). After rinsing, the sections were incubated with ready-to-use primary antibodies, with peroxidase activity blocked beforehand. Subsequently, the primary antibodies were detected using a secondary antibody at room temperature. The sections were then treated with the avidin-biotin complex for 10 minutes at room temperature and visualized using diaminobenzidine (DAB) as the chromogen.

Finally, all sections were counterstained with hematoxylin, dehydrated, and mounted for analysis.

#### Interpretation

Immunohistochemical staining was independently evaluated by two senior pathologists. Only membranous and/or cytoplasmic staining was considered positive. CD25+ and CD3+ TILs were assessed in two distinct locations: the intra-tumoral (IT) region and the peri-tumoral (PT) region. The peri-tumoral region was defined as the invasive margin in surgical specimens. For biopsy samples, the peritumoral region was considered as the stroma surrounding the tumor nests.

For each location, five consecutive high-power fields (HPFs) were selected, and TILs were manually counted. The final density of each lymphocyte population in each region was calculated as the mean count across the five selected HPFs. TIL densities were then categorized as low or high, using the median value of each marker as the cut-off.

### Statistical Analysis

Follow-up time was defined as the duration in months from the date of primary diagnosis to the last known contact or death. Overall survival (OS) was calculated as the time in months from the initial diagnosis to the last known contact or death. Event-free survival (EFS) was defined as the duration in months from the primary diagnosis to the first occurrence of progression, recurrence, or metastases.

Qualitative variables were summarized as frequencies and percentages, while quantitative variables were expressed as medians and ranges. The association between TIL densities and clinicopathological parameters was analyzed using the chi-square test or Fisher’s exact test, as appropriate. Kaplan-Meier survival analysis and the log-rank test were used to compare patient survival curves.

Variables with a p-value ≤ 0.2 in univariate analyses were included in the multivariate Cox regression model to identify independent predictors of survival, balancing model complexity with our sample size. A p-value of less than 0.05 was considered statistically significant. All statistical analyses were conducted using SPSS Statistics software (version 21).

## RESULTS

### Patients’ Characteristics and Evolution

Out of 35 retrieved cases, 30 cases met the inclusion criteria and were included in the study. Tumor samples were obtained from 18 TURBTs and 12 surgical specimens. The median age of patients was 65 years (range: 42–90), with a male-to-female ratio of 9:1. Multiple tumors were observed in 33% of cases, and the median tumor size was 45 mm.

High-grade UC was the most frequent diagnosis, with histological variants identified in 11 cases (37%). Lympho-vascular invasion was present in 57% of the cases, and associated ISC was observed in 17% of the cases. Muscle-invasive tumors (≥ pT2) accounted for 70% of the cases, and lymph node involvement was noted in 43%.

Clinicopathological features of the cohort are detailed in Table 1.

**Table 1:** Patient characteristics

	**All cases n=30 (%)**
Age (Median) 65 years (range 42-90)	
Gender	
Male	27 (90)
Female	3 (10)
Tumor size	
≤45mm	8 (27)
>45 mm	8 (27)
Unavailable	14 (46)
Multifocality	
Presence	19 (63)
Absence	10 (33)
Unavailable	1 (4)
Histological type of urothelial carcinoma	
Classical	19 (63)
Histological variant	11 (34)
In situ Carcinoma	
Absence	25 (83)
Presence	5 (17)
Lymphovascular invasion	
Absence	13 (43)
Presence	17 (57)
Tumor stage	
pT1	9 (30)
≥pT2	21 (70)
Lymph node involvement	
Absence	12 (40)
Presence	13 (43)
Unavailable	5 (17)

The mean follow-up time was 33 months. During this period, seven patients experienced tumor progression and/or recurrence, and nine developed distant metastases. The mortality rate was 50%. The median overall survival (OS) and event-free survival (EFS) were 18 months and 26 months, respectively.

### Immunohistochemistry Analysis

All cases showed positive staining in both analyzed locations. The distribution of TILs varied heterogeneously across patients in each region. CD3+ (p=0.035) and CD25+ (p=0.051) TILs were more frequently observed in the stromal compartment.

**Figure 1:**
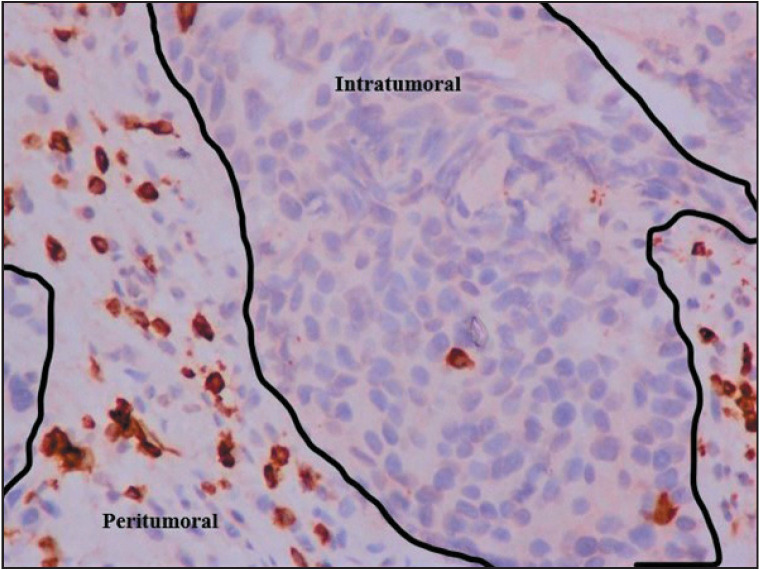
High density of peritumoral CD3+ TILs and low density of intratumoral CD3+ TILs (IHC x 40)

The median CD3+ TIL count was 14 cells/mm² (range: 2–136 cells/mm²) in the tumor core and 56 cells/mm² (range: 6–160 cells/mm²) in the peri-tumoral region. High CD3+ TIL densities were observed in 47% of cases in the intra-tumoral region and in 50% of cases in the peri-tumoral region (Figure 1).

**Figure 2:**
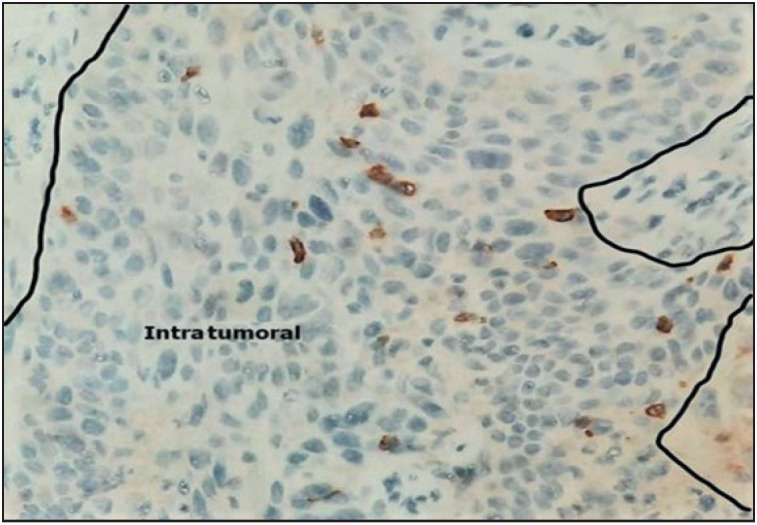
High density of intratumoral CD25+ TILs (IHC x 40)

The median CD25+ TILs count was 2 cells/mm² (range: 0–21 cells/mm²) in the tumor core and 6 cells/mm² (range: 0–26 cells/mm²) in the peri-tumoral region. High CD25+ TIL densities were identified in 37% of the cases in the intra-tumoral region (Figure 2) and in 43% of the cases in the peri-tumoral region.

A summary of CD3+ and CD25+ TIL densities is provided in Table 2.

**Table 2:** Median TILs densities and categorization into Low and High groups

**TILs density**		**Median count (cells/mm2)**	**Category**	**All cases n=30 (%)**
CD3+ TILs	Intratumoral	14	Low	16 (53)
			High	14 (47)
	Peritumoral	56	Low	15 (50)
			High	15 (50)
CD25+ TILs	Intratumoral	2	Low	19 (63)
			High	11 (37)
	Peritumoral	6	Low	17 (57)
			High	13 (43)

### Relationship Between the Clinicopathological Parameters, TILs and Ratio, and Survival

CD25+ and CD3+ TILs densities were not significantly associated with clinicopathological features. Kaplan-Meier survival analysis revealed a significant association between the presence of carcinoma in situ (p=0.050), distant metastases (p=0.042), high pathological tumor (pT) stage (p=0.001), and poor OS. A significant relationship was also observed between high pT stage (p=0.002) and shorter event-free survival (EFS).

**Figure 3:**
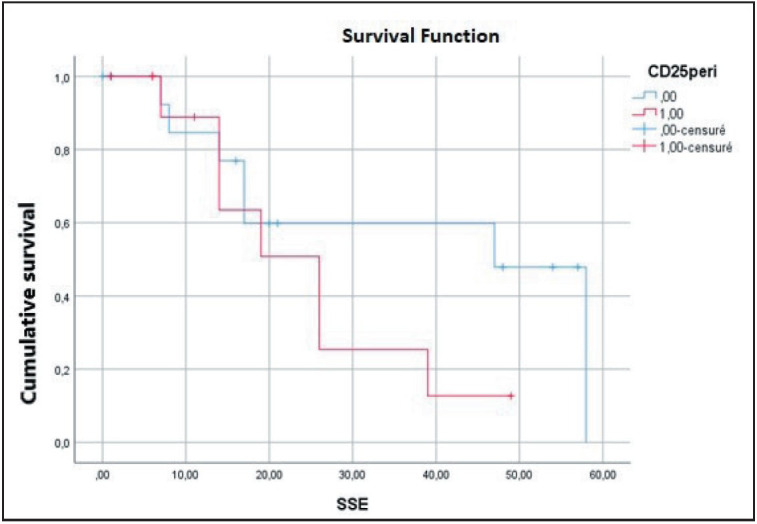
Overall survival according to in situ carcinoma

**Figure 4:**
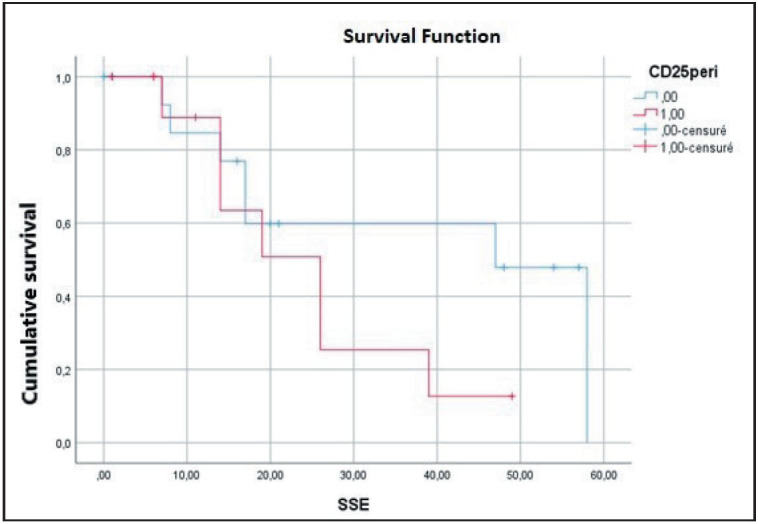
Overall survival according to stage

**Figure 5:**
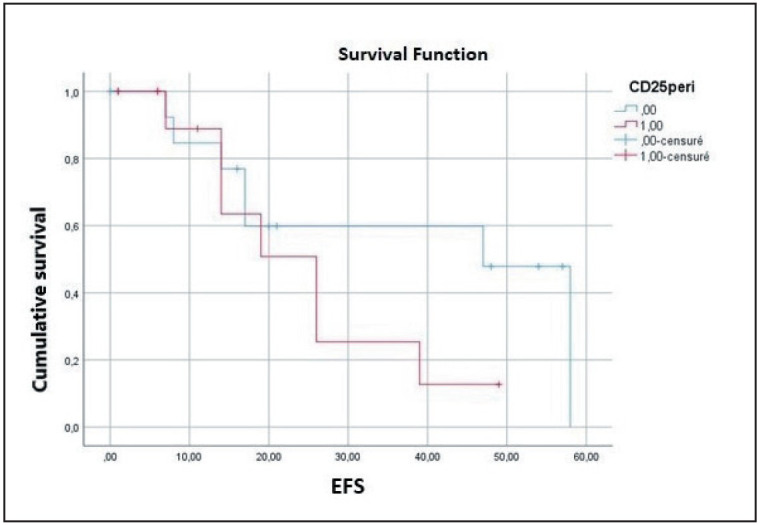
Disease free survival curve according to peri-tumoral CD25 TILs

Multivariate analysis identified ISC (p=0.040) and high pT stage (p=0.004) as independent predictors of OS. Additionally, peri-tumoral CD25+ TIL density (p=0.052, almost significant) and tumor stage (p=0.041) were independent predictors of EFS (Figure 3, Figure 4, Figure 5).

The results of the univariate and multivariate analyses are summarized in Table 3 and Table 4.

**Table 3:** Univariate analysis of TILs role in predicting overall and disease-free survival

		**Median of overall survival** **(CI at 95%)**	**p**	**Median of disease-free survival** **(CI at 95%)**	**p**
Intratumoral CD3+ TILs	Low	20 (18.5 – 21.4)	0.791	19 (0 – 42)	0.558
	High	47 (6.7 – 87.3)		26 (8.6 – 43.3)	
Peritumoral CD3+ TILs	Low	20 (8.1 – 31.8)	0.486	17 (12.5 – 21.4)	0.502
	High	47 (0 – 107)		39 (7.3 – 70.6)	
Intratumoral CD25+ TILs	Low	20 (0 – 54.6)	0.759	39 (7.2 – 70.7)	0.135
	High	26 (15.2 – 36.7)		14 (3 - 25)	
Peritumoral CD25+ TILs	Low	20 (0 – 69)	0.564	47 (26.1-67.8)	0.198
	High	26 (17 – 35)		26 (16.4 – 35.5)	
Intratumoral CD25/CD3 ratio	Low	17 (6 – 28)	0.511	39 (6.5 – 71.4)	0.674
	High	26 (0 – 63)		19 (7 – 31)	
Peritumoral CD25/CD3 ratio	Low	20 (0 – 60.2)	0.785	26 (5.6 -46.4)	0.829
	High	26 (16 – 36)		26 (5.7 – 46.2)	

**CI:** Confidence interval

**Table 4:** Multivariate analysis of factors predicting overall and disease-free survival

	**Overall survival**			**Disease-free survival**		
	**HR**	**CI**	**p**	**HR**	**CI**	**p**
In situ Carcinoma	3.53	1.03 -12.06	**0.040**	-		
Tumor stage	2.26	1.29 - 3.94	**0.004**	3.16	1.16 - 8.58	**0.021**
Peritumoral CD25+ TILs	-			4.06	0.97 -17.03	**0.052**

**HR: **Hazard  Ratio, **CI:** Confidence interval

## DISCUSSION

Our study demonstrated that CD3+ and CD25+ TILs are present in all tumors, both at the tumor core and in the peritumoral (PT) region, with a higher frequency in the stroma. Through the phases of immune surveillance and equilibrium in immunoediting, the immune system typically constrains tumor growth. However, tumor subclones with reduced immunogenicity may escape immune detection by various mechanisms, including the recruitment of Treg cells (7). Previous studies have shown that all immune cell types, including Tregs, are found within the tumor microenvironment, predominantly at the tumor core, invasive margin, and in tertiary lymphoid structures (8). The recruitment of Tregs from the circulation signifies the establishment of an immunosuppressive TME in bladder cancer (11), with Tregs often concentrated in the stroma and tertiary lymphoid structures (12). Our findings align with these observations. The overall immune infiltration varies between tumor histological types, patients, and even within different areas of the same tumor (12). The reasons for intra- and intertumoral heterogeneity are not yet fully understood. A recent study highlighted that the immune composition of the TME in MIBC differs between molecular subtypes (13). Furthermore, the diversity of immune cell infiltration appears to be orchestrated by a chemokine network, driven by tumor-associated antigens, which creates varying immune compositions both within individual tumors and between different tumors (7,12,14).

The clinical impact of immune contexture has been explored in various tumor types, yielding heterogeneous results (8,12). In ovarian, breast, and lung cancers, a high density of Tregs has been associated with poor survival and relapse (9,10,15), while in colorectal cancer, high Treg infiltration correlated with improved survival (16). In bladder cancer, data is limited and conflicting (4,17). In our study, CD25+ and CD3+ TILs were not significantly associated with clinicopathological features. However, multivariate analysis showed that a high density of regulatory CD25+ T lymphocytes (p=0.052) predicted worse event-free survival. Our results are consistent with those of Murai et al. who found high peri-tumoral Treg density to be predictive of poor DFS (18). In contrast, two previous studies reported that high intra-tumoral Treg density was associated with prolonged OS and DFS, while others found no correlation between Treg density and outcome (19–22).

Some studies have focused on the proportion of Tregs among effector cells. High Treg/CD3, Treg/CD8 ratios, and an inverted T effector cell/Treg ratio have been linked to poor OS and recurrence (23,24). The disparity in these results complicates interpretation and may be due to several factors such as: the differences in inclusion criteria (treatment, stage), the tumor sample types (biopsies vs. surgical specimens), and the Treg quantification methods. Most previous studies used IHC; however, IHC is not standardized in bladder UC, and there is no consensus on a cut-off value for categorizing Treg densities as low or high (17). Additionally, Tregs were identified using different phenotypic markers, such as CD25 and Forkhead box protein P3 (FOXP3) (12). Indeed, most studies used FOXP3 to identify Tregs, but CD25 and FOXP3 are not exclusive to Tregs and can be upregulated in some effector T cells, including cytotoxic lymphocytes (25). This may explain the correlation between high FOXP3+ cell density and better outcomes in bladder cancer, as reported by Winerdal et al. (19). Thus, combining the CD4, CD25, and FOXP3 immunohistochemical markers is likely the most effective approach to identify Tregs (25).

Although contradictory results exist, it is difficult to draw firm conclusions regarding the prognostic value of Tregs. Nevertheless, Tregs seem to be predictive of poor outcomes, and our findings are consistent with this. However, the limitations of our study include its retrospective design, limited sample size, and reliance on a single marker to identify Tregs.

Beyond their prognostic value, Tregs also appear to predict treatment response in bladder UC. An increased number of Tregs has been associated with BCG therapy failure and recurrence in previous studies (26). In the study published by Baras et al. the authors found that Treg density alone was not predictive of response to cisplatin-based neoadjuvant CT, but the CD8/Treg TIL ratio was strongly associated with response. Patients with a CD8/Treg ratio <1 did not respond to CT (27). These findings suggest that not the density of Tregs but their proportion among total T cells may be prognostic in BC (4).

In the era of immune checkpoint inhibitors, TILs represent promising prognostic and predictive biomarkers, and they may also serve as therapeutic targets. Treg depletion combined with checkpoint inhibitors could offer potential synergistic effects. Recent evidence has shown that tumor-infiltrating Tregs have a distinct cell surface phenotype, making them promising molecular targets to integrate into such therapeutic combinations (28).

## CONCLUSIONS

In conclusion, our study demonstrated that all tumors were infiltrated with CD3+ and CD25+ TILs, and that regulatory T cells may serve as predictors of recurrence in urothelial carcinoma of the bladder. These promising findings highlight the necessity for larger studies to better define the clinical impact of Tregs in BC. Moreover, the establishment of a standardized IHC method for identifying Tregs, along with the definition of cut-off values for categorizing their densities, could facilitate the incorporation of this parameter into pathological reports as a new prognostic factor. This approach could improve risk stratification and inform therapeutic decision-making. Finally, Tregs represent an attractive therapeutic target, with the potential to significantly alter the management of BC.

## Conflict of Interest

No potential conflict of interest was reported by the author(s).

## Software Availability Statement

The statistical analyses performed in this article using SPSS21 software can be conducted using the freely accessible software Jamovi https://www.jamovi.org . The user manual is available at the following link. https://lsj.readthedocs.io/ru/latest/Ch03/Ch03_jamoviIntro_1.html
